# 

**Published:** 1918-05-04

**Authors:** 


### Manager's Notices.

**Now Ready, "The Hospital" Index to Vol. LXIII., Oct. 1917 to March 1918. Price 3½d. per copy post free.**

**RATES OF SUBSCRIPTION (payable in advance).**

| | |
|---|---|
| UNITED KINGDOM. | |
| Three Months (including Postage) ... | 2s. 0d. |
| Six Months do. ... | 4s. 0d. |
| Twelve Months do. ... | 6s. 6d. |
| FOREIGN AND COLONIAL. | |
| Three Months (including Postage) ... | 2s. 6d. |
| Six Months do. ... | 5s. 0d. |
| Twelve Months do. ... | 8s. 8d. |

**All letters on business matters must be addressed to The Manager, "The Hospital," 28 and 29 Southampton Street, London, W.C. 2.**

**Special Rates** will be quoted to those institutions that agree to send all their public notices to THE HOSPITAL, so as to make it a file of such announcements for ready reference.

### Forthcoming Events.

### The Royal Institute of Public Health.

A SERIES of lectures on "Public Health Problems under War and after War Conditions" will be held on the Wednesdays in May, June, and July, at 4 P.M., at the Lecture Hall of the Institute, 37 Russell Square, W.C.1.

### National Baby Week Campaign.

A COURSE of six lectures to prospective speakers and others interested in the Baby Week campaign will be held on Mondays, May 6 to June 10, at 5.30 P.M., at 14 Gordon Square, W.C. Cards of admission can be obtained free on application to the Secretary, National Baby Week Council, 27A Cavendish Square, W. 1.

THE HOSPITAL FOR SICK CHILDREN, GREAT ORMOND STREET, W.C. 1.—Board Room of the Hospital, Wednesday, May 15, at 4.30 P.M. precisely.

### St. Chad's Hospital, Birmingham.

On the occasion of the retirement of the matron (Miss M. Cotton) the members of the medical staff of St. Chad's Hospital, Birmingham, met and expressed their appreciation of the untiring and successful efforts made by the matron on behalf of the hospital since its opening, some three and a half years ago. A tribute was paid Miss Cotton for the standard of excellence in nursing which the probationers trained under her guidance had attained, as evidenced by the Medical Examiners at the last annual test. A presentation of a purse containing a substantial cheque was made to Miss Cotton by the Chairman.

